# Thoughts on Self-Harm in Polish Pregnant and Postpartum Women During the Pandemic Period

**DOI:** 10.3390/jcm13216449

**Published:** 2024-10-28

**Authors:** Urszula Sioma-Markowska, Róża Motyka, Patrycja Krawczyk, Karolina Waligóra, Anna Brzęk

**Affiliations:** 1 Department of Nursing in Gynaecology and Obstetrics of Women Health Division, Faculty of Health Sciences in Katowice, Medical University of Silesia in Katowice, Medyków 12 Street, 40-752 Katowice, Poland; urszula.markowska@sum.edu.pl (U.S.-M.); roza.motyka@sum.edu.pl (R.M.); pkrawczyk@sum.edu.pl (P.K.); kwaligora@sum.edu.pl (K.W.); 2 Department of Physiotherapy, Faculty of Health Sciences in Katowice, Medical University of Silesia in Katowice, Medyków 12 Street, 40-752 Katowice, Poland

**Keywords:** pregnancy, postpartum, depression, self-harm thoughts, EPDS, risk factors

## Abstract

**Background**: The risk of depression during pregnancy and postpartum is high and has intensified during the COVID-19 pandemic. The aim of this study was to estimate the risk of depressive disorders and self-harm thoughts in the third trimester of pregnancy and the first week postpartum in the pandemic period. **Methods**: This study involved a total of 317 pregnant and postpartum women. The risk and severity of depressive disorders and the prevalence of self-harm thoughts in women during the perinatal period were assessed using EPDS. **Results**: Pregnant women were significantly more likely to have higher EPDS scores compared to postpartum women. Mild depressive symptoms were reported by 22.08% of pregnant women and 17.18% of postpartum women, and severe symptoms were observed in 25.97% of pregnant women and 16.56% of postpartum women. Thoughts of self-harm were reported by 11.69% of pregnant women and 17.79% of postpartum women. Self-harm thoughts were significantly more common among pregnant women: multiparous women, pregnant women who received psychiatric treatment before pregnancy, those diagnosed with depressive disorders, those who suffered from prolonged periods of anxiety and sadness, and those lacking a supportive person. Among postpartum women, there were statistically significant differences in the prevalence of self-harm thoughts for place of residence, education, type of occupation, number of pregnancies, course of pregnancy, and presence of a supportive person. **Conclusions**: The increased prevalence of depressive symptoms and self-harm thoughts related to the COVID-19 pandemic highlights the urgent need for screening among pregnant women and the implementation of clinical interventions.

## 1. Introduction

Antenatal and postnatal depression is a serious mental health disorder in the perinatal period. Unfortunately, the pathophysiology of this condition is not fully understood, which makes establishing appropriate diagnosis very difficult [1]. Suicide and suicide attempts in pregnant women are less common, relative to the general female population, but in the postpartum period, suicide is the cause of death for about 20% of women [2]. In addition, thoughts of self-harm during pregnancy affect between 5% and 16.8% of women [3,4], and the presence of suicidal thoughts during the first months of pregnancy is estimated at 13.1% to as high as 33% [5]. This is especially true for young women who do not have partners or family support.

Self-harm is a serious health problem that can range from self-harm ideation to actual acts of self-harm or behavior resulting in injury or death. In the 20–24 age group, self-harm causes 10.1% of deaths [6]. Self-harm ideation can adversely affect both the fetus and mother. A 2019 cohort study showed that self-harm during pregnancy was linked to increased depressive symptoms and a worse mother–child relationship [7]. Thoughts of self-harm are a significant predictor of suicidal thoughts, which can lead to suicide attempts or suicide deaths. Hence, the importance of identification, prompt intervention, and proper care. 

The primary tool used to assess the risk of postpartum depression, also recommended during pregnancy, is The Edinburgh Postnatal Depression Scale (EPDS) [8]. 

Mood disorders during pregnancy can prognosticate postpartum depression. According to one study, a high score on the EPDS obtained at 32 weeks’ gestation correlates with a high, depression-indicating score on a postpartum test [9]. The risk of depression during and after the pregnancy or the worsening of its symptoms is high, and cases of depression especially among pregnant women are often not diagnosed early enough. In extreme cases, pregnancy depression can lead to suicidal behavior.

The COVID-19 pandemic caused significant public health threats and increased levels of fear, anxiety, and uncertainty, particularly among pregnant women [10], and meeting the mental health needs of pregnant and postpartum women became a major challenge [11]. The prevalence of depression in pregnant and postpartum women increased during the COVID-19 pandemic, ranging from 17% to 31% [10,12,13]. In the literature, many meta-analyses can be found addressing mental health, with a particular focus on depression and anxiety in pregnant and postpartum women during the pandemic. Unfortunately, only a few studies have highlighted the issue of self-harm thoughts during pregnancy and early postpartum. These studies mainly focus on the USA and China [14,15], which makes comparison to Europe difficult. The authors, after conducting their research, hope to fill this gap in the literature, and the results may become an important element of preventive actions in the discussed area in the future.

The aim of this study was to estimate the risk of depressive disorders in the third trimester of pregnancy and the first week of the postpartum period and the frequency of self-harm thoughts during the COVID-19 pandemic, along with an attempt to explain their determinants, taking into account selected risk factors. 

## 2. Materials and Methods

This study was a cross-sectional observational study. The sample size was calculated by the Raosoft program (2007), assuming a 95% confidence level, margin of error of 5%, and population size of 20,000. This analysis indicated a recommended sample size of 377. Out of more than 400 qualified women, some did not correctly complete the questionnaires, some were incomplete, and a few women withdrew due to feeling unwell. The lack of fully completed questionnaires was considered an exclusion criterion. 

A total of 317 pregnant and postpartum women were included in the final analysis. The women differed in age, material status, education, marital status, and obstetric history. The participants came from the Silesia region, with 73% coming from large cities and only above 25% from rural areas (35% in pregnancy group and 14% in postpartum group). Over 55% of the women surveyed had higher education, while only 5% had primary education. A detailed description is presented in Table 1 and outlined in the Section 3. This study was conducted between March 2021 and March 2022. Pregnant women in the third trimester of pregnancy (*n* = 154) were classified into the “pregnancy” group, and postpartum women (*n* = 163) who were between 1 and 7 days postpartum were classified into the “postpartum” group. 

The Edinburgh Postnatal Depression Scale (EPDS) was used to assess the risk and severity of depressive disorders and the occurrence of self-harm thoughts in women during the perinatal period. The researchers used their own questionnaire to collect sociodemographic and clinical (obstetric interview) data.

The study design was approved by the Bioethics Committee of the Medical University of Silesia in Katowice (PCN/CBN/0022/KB/283/21). The research was conducted as part of statutory research. 

The EPDS includes 10 questions on anhedonia, blaming, anxiety, fear, feeling of being overwhelmed, sleep problems caused by sadness, grief, crying, and thoughts of self-harm. Each statement is scored on a scale of 0–3 points. The maximum number of points is 30, with 10 points or the reply confirming a desire to self-harm taken as the lower limit for the likelihood of depression. Postpartum women should complete the EPDS during the first week after delivery, preferably on the third day postpartum, as research indicates that a high EPDS score at this time, even in women who do not report depressive symptoms, is associated with a high risk of developing full-blown postpartum depression within the next six weeks [8]. Low self-esteem is one of the essential symptoms of postpartum depression. An affirmative answer to question No. 10 (“*it happened that I thought about hurting myself*”) on the EPDS form is considered diagnostic. Even if the total score obtained on the EPDS is not high but includes any positive (scored) answer to question 10, the interpretation should be that the woman requires psychiatric consultation as a matter of urgency.

The questionnaires were completed by the women independently, with the option to seek advice from an expert online. At any point, the participant could clarify a question and resolve any doubts. This approach allowed for a sense of anonymity on one hand, while ensuring the research was conducted accurately on the other.

Statistical analyses were carried out using the Excel and Statistics 12v programs. Descriptive statistics of the studied parameters—medians (Me), means (X), standard deviations (SD)—were performed. Non-parametric statistics were used for intergroup comparisons: the Man–Whitney U test for two groups and Chi-squared test with Yates correction. The Shapiro–Wilk test was used to evaluate the normal distribution. Missing data analysis and collinearity diagnostics were conducted before the analyses. The assumed level of statistical significance was *p* < 0.05. 

## 3. Results

The mean age of pregnant women participating in this study was 28.9 years, ±3.7 (Me 29.0), while the mean age of the postpartum women was 29.9 ± 5.6 (Me 29.0). The average gestational age in the third trimester was 36.1 ±1.8 weeks, and at delivery it was 38.4, ±2.1 weeks. The statistically significant differences were observed for the place of residence (*p* = 0.00005), education (*p* < 0.00001), type of occupation (*p* = 0.02), and number of pregnancies (*p* = 0.0001). Pregnant women were dominated by primiparas (68.83%) and postpartum women were dominated by multiparas (53.37%). 

Pregnant women were significantly more likely to respond to a question about psychiatric treatment before pregnancy (*p* = 0.0007) and the presence of prolonged periods of anxiety and sadness (*p* = 0.01). Of the subjects, 13.31% were diagnosed with depressive disorders before pregnancy. Further, 10.39% of pregnant women and 4.91% of postpartum women experienced a lack of support from their husband/partner. The characteristics of the study groups including sociodemographic and medical variables are shown in Table 1 and Table 2.

Pregnant women were significantly more likely (*p* = 0.005) to have higher EPDS scores compared to postpartum women (Table 3). The absence of depressive symptoms was observed in 80 (51.95%) pregnant women and 108 (66.26%) postpartum women. Mild depressive symptoms (≥10–13 pkt EPDS) were reported by 34 pregnant women (22.08%) and 28 (17.18%) postpartum women, and severe symptoms were observed in 40 (25.97%) pregnant women and 27 (16.56%) postpartum women (*p* = 0.03) (Table 4). In this study, the EPDS achieved a high α-Cronbach’s reliability index value of 0.922 for the “pregnancy” group and 0.877 for the “postpartum” group.

Table 5 shows the distributions of responses to question 10 of the EPDS form. There were no statistically significant differences in these distributions (*p* = 0.17). Thoughts of self-harm were reported by 47 women (14.96%), including 18 pregnant women (11.69%) and 29 (17.79%) postpartum women. 

Table 6 presents the numbers of women who gave affirmative answers to EPDS question No. 10 on thoughts of self-harm, depending on sociodemographic factors and obstetric history data. The reported numbers were correlated by percentage to the number of women in the category in the group. The table also includes the results of a test comparing the frequency of thoughts of self-harm both within each group and between groups for a given category.

Among women living in the city, 14 pregnant women (14%) and 19 (13.67%) in the postpartum period reported thoughts of self-harm. The same thoughts were reported by 4 (7.41%) pregnant women and 10 (41.67%) postpartum women living in rural areas (*p* = 0.0009). Postpartum women with higher education (25.00%) or who performed physical labor (29.55%) showed thoughts of self-harm significantly more often (*p* = 0.005; *p* = 0.03) compared to pregnant women (8.33%; 6.67%) (Table 6).

Among pregnant women, statistically significant differences were found in the prevalence of thoughts of self-harm for the number of pregnancies and answers to obstetric interview questions. Self-harm ideation was significantly more common among multiparous women (*p* = 0.04). Self-harm thoughts were also more common in pregnant women who had received psychiatric treatment prior to pregnancy (*p* = 0.04), who had been diagnosed with depressive disorders (*p* = 0.05), and who had experienced prolonged periods of anxiety and sadness (*p* = 0.0001). The absence of a supportive person showed a statistically significant effect on the frequency of thoughts of self-harm (*p* < 0.00001). 

The group of postpartum women showed statistically significant differences in the prevalence of thoughts of self-harm for the following sociometric factors: place of residence (*p* = 0.03), education (*p* = 0.04), and type of occupation (*p* = 0.01). Obstetric history showed that the number of pregnancies (*p* = 0.04), the course of pregnancy (*p* = 0.04), and the presence of a supportive person (*p* = 0.001) had a statistically significant influence on the prevalence of self-harm ideation. Further, 75% of women lacking a supportive person had thoughts of self-harm.

The comparison of differences in the prevalence of thoughts of self-harm between groups across sociometric factors showed statistically significant results for: rural residence (*p* = 0.009), higher education (*p* = 0.005), and manual labor (*p* = 0.03). Obstetric history showed significant differences for primiparas (*p* = 0.002), women who did not receive psychiatric treatment prior to the current pregnancy (*p* = 0.05), and for women who did not experience prolonged periods of anxiety and sadness (*p* = 0.004) (Table 6). 

## 4. Discussion

Our study conducted using the EPDS showed that depressive disorders were significantly more common in pregnant women compared to women in the postpartum period in the pandemic period. High depression scores (≥10 points) were obtained in women in the third trimester of pregnancy in just under half of pregnant women and in over one-third of postpartum women in the first week of postpartum. EPDS is considered a good screening tool [16] for identifying women who need a thorough psychosocial and clinical evaluation. There are no population-based epidemiological studies of perinatal depressive disorders being carried out in Poland. According to World Health Organization estimates, globally, about 10% of pregnant women and 13% of postpartum women experience mental disorders, mainly depression [17]. Increasingly, however, postpartum depression is being analyzed as a variable in cross-sectional studies. Studies conducted prior to the COVID-19 pandemic showed that the percentage of women experiencing depressive symptoms during pregnancy and the postpartum period was similar to the prevalence rates of pregnancy and postpartum depression in the world population. In a 2014 Polish study, depressive symptoms in the third trimester of pregnancy affected 14% of the women surveyed [18]. In another Polish study, 35% of women obtained a high score of 12 or more on the postpartum depression scale [19]. In contrast, Fejfer-Szpytko et al. estimated the prevalence of postpartum depression at 10% to 30% [20].

Antenatal depression is considered a significant predictor of depression in the postpartum period. Global recommendations, as well as the Polish Ministry of Health’s 2019 recommendation, emphasize the importance of conducting early screening for postpartum depression (MZ 2019; RNAO 2019; USPSTF 2019; ACOG 2018; NICE 2018; EU 2016; BC Mental Health 2014; NICE/NCCMH 2014) [21]. EPDS is recommended as the best screening tool dedicated to identifying potential pregnant and postpartum women with depression (MZ 2019; AGDH 2018; BC Mental Health 2014; NICE 2018). All pregnant women and those in the postpartum period for up to one year should be covered by screening (MZ 2019; RNAO 2019; USPSTF 2019; AGDH 2018; ACOG 2018; NICE 2018; EU 2016; BC Mental Health 2014; NICE/NCCMH 2014), with special attention paid to women with additional risk factors (including a history of depression, experience of sexual harassment or violence) (MZ 2019; NICE 2018; NICE/NCCMH 2014) [21]. Training should be provided to medical personnel, with a particular focus on midwives caring for pregnant women, involving recognizing postpartum depression and using screening tools [22]. 

Polish recommendations indicate that the examination of a woman’s mood in each case should be supplemented by an interview that includes psychological and social aspects (Ministry of Health 2019, Polish Midwifery Association 2019) [23,24]. Postpartum women should fill out the EPDS in the first week after delivery—preferably on the third postpartum day—as research indicates that a high score on the EPDS at this time, even in women who do not report depressive symptoms, is associated with a high risk of developing full-blown postpartum depression over the next six weeks [25].

The UK’s National Institute for Health and Care Excellence/National Collaborating Centre for Mental Health (NICE/NCCMH 2014) stresses that in the event of a positive score (i.e., 1, 2, or 3 points) on question 10 (relating to thoughts of self-harm), immediate consultation with a specialist (psychiatrist, clinical psychologist) and referral for psychiatric treatment is necessary [26]. In a situation where this disorder is suspected, all existing sources recommend psychiatric consultation as a matter of urgency.

These regulations and recommendations, however, pertain to the period before the pandemic and the effects it has caused, particularly in the group of pregnant and postpartum women. These regulations should take into account the current epidemiological situation in the country and the world and be modified accordingly. The results of pre-pandemic studies are alarming.

The COVID-19 pandemic caused significant public health threats and increased fear, anxiety, and uncertainty, particularly among pregnant women who feared infection and its health consequences for their pregnancy and, in the case of postpartum women, their newborns [10]. Numerous studies, statistical reviews, and meta-analyses conducted by researchers show that the overall prevalence of depression in pregnant and postpartum women during the COVID-19 pandemic was 25% [27], and they clearly emphasize that the pandemic period intensified depression in pregnant and postpartum women. According to Adrianto et al., approximately one-third of women experienced depression [11], similar to findings in other studies [28].

Additional factors exacerbating symptoms included difficult economic conditions, loss of income, insufficient social support, and lack of access to healthcare professionals [29,30,31,32].

While the results of this study on the prevalence of postpartum depression are comparable to those of other authors, the high percentage of women with depressive symptoms during pregnancy is alarming.

A recent study involving 2839 pregnant women in their third trimester from ten provinces in China found a prevalence of depression of 26.0% [15]. Although mental health issues were recognized in all countries during the pandemic, and crisis psychological intervention guidelines specifically for COVID-19 were published, for example, by the National Health Commission of the People’s Republic of China, perinatal women were not singled out in these guidelines as a vulnerable population [33].

In this study, thoughts of self-harm were declared by 11.69% of pregnant women (third trimester of pregnancy) and 17.79% of postpartum women in the first week after delivery. Although no statistically significant differences in these distributions were shown, a positive answer to a question about the desire to self-harm in the EPDS is considered a lower bound for the likelihood of postpartum depression. Cantwell R et al. claim that the majority of suicides among pregnant and postpartum women (approximately 60%) occur during the 6 weeks before delivery and 12 weeks after birth [34].

Screening for self-harm ideation and suicidal thoughts among pregnant women can be conducted with no additional visits or extra costs. Focusing on suicidal thoughts, regardless of depression, is important. This study showed that suicidal thoughts without clinically detectable mental health disorders are common [35]. Yanting Wu’s research clearly indicated an increase in the percentage of women with self-harm thoughts, which could potentially result in death and injuries indirectly caused by the COVID-19 outbreak. The authors emphasize that perinatal mental health interventions should be a priority during any widespread epidemic and should be a focus during an international public health crisis [15].

Thoughts of self-harm are an important indicator of mental health risk and the risk of attempted and successful suicide. A study by Redinger et al. [36] showed that the risk of developing thoughts of self-harm begins early in pregnancy. The prevalence of thoughts of self-harm was estimated at 12.5% in early pregnancy and 11.6% in late pregnancy. In contrast, in a group of Chinese pregnant women (*n* = 898), the prevalence of antenatal depression was 24.4%, and 12.8% of women showed thoughts of self-harm (EPDS assessment) with pregnant women in different trimesters reporting a similar prevalence of self-harm thoughts [37].

There are few scientific studies on the increase in self-harm thoughts during the pandemic, and those that are available pertain to the period before the pandemic, highlighting a serious issue [38,39,40]. Our study fills this gap, emphasizing the importance of the problem. The results showed that postpartum women who were rural residents significantly more often compared to urban residents, and significantly more often compared to pregnant women who were rural residents declared experiencing self-harm ideation. Other socioeconomic variables that significantly more often influenced the occurrence of thoughts of self-harm in postpartum women were higher education and labor work. The cross-sectional study conducted by Liu et al. indicated that losing a family member due to COVID-19 and worries about receiving financial support were positively associated with thoughts of self-harm. Interestingly, working remotely from home was inversely correlated with thoughts of self-harm [14].

This study showed that self-harm thoughts were significantly more prevalent in a group of pregnant women who were multiparous, with a history of a psychiatric treatment, diagnosed depression or prolonged periods of anxiety and sadness, or without a supportive person. In contrast, in the postpartum group of women, variables which significantly more likely predisposed to thoughts of self-harm were childbearing (primiparas), complicated delivery, and lack of support from loved ones. Among postpartum women who lacked social support, as many as 75% declared thoughts of self-harm.

### Summary—Practical Implications, Limitations

Pregnant women with a history of mental health disorders are in a high-risk group and should be closely monitored for symptoms of depression and suicidal ideation during pregnancy and for a year after delivery. Every pregnancy is different, even for the same woman, and women’s multiple roles in work and family life are significant causes of mental health disorders in pregnancy. Women who do not have social support or come from families with low socioeconomic status also pose a risk of developing prenatal and postnatal depression and suicidal thoughts. There is a need to conduct a routine assessment of the presence of depressive symptoms and self-harm ideation using simple screening tools in every pregnant and postpartum woman.

This study had some limitations. First, it was carried out during the pandemic period, hence the high EPDS scores; thus, any generalization of conclusions should be done with caution. Second, the EPDS self-assessment scale used in this study is recommended for assessing perinatal well-being and depressive symptoms, but has some limitations in distinguishing depression from other mental disorders. The next phase could be conducting a multi-center study that would include more time points for measuring mental status during pregnancy and one year after delivery. The presented results are part of a larger project. In this paper, the focus was primarily on highlighting an existing, rarely described issue of self-harm during pregnancy and postpartum. In further studies, the authors will focus on dividing the women in each of the studied groups into those diagnosed with depression and those without such a diagnosis. This division will allow for a more comprehensive presentation of the issue, which could be considered a limitation of this paper. The more significant limitation is the sample size; however, it is important to clearly emphasize the timing of the research, conducted during the pandemic, which posed much greater challenges in accessing the studied groups of women, especially considering that this study was conducted in the presence of a researcher. 

## 5. Conclusions

Our results indicate a clinically significant increase in the prevalence of depressive symptoms during the third trimester of pregnancy and self-harm thoughts in the early postpartum period during the pandemic. In addition to the well-documented perinatal mental health risk factors, we found that first-time mothers, younger women, those with higher education, living in rural areas, and performing manual labor were more prone to experiencing self-harm thoughts during the postpartum period. Multiparous women without support from loved ones and with a history of depression were at risk of self-harm during pregnancy. Our study indicates a significantly increased prevalence of depressive symptoms, self-harm thoughts, and anxiety symptoms related to the COVID-19 pandemic, highlighting the urgent need for screening among pregnant women, followed by clinical interventions and consistent prevention and treatment of these conditions.

## Figures and Tables

**Table 1:** Characteristics of the studied groups—sociodemographic variables.

Factor	Category	“Pregnancy” Group (*n* = 154; 100%)	“Postpartum” Group (*n* = 163; 100%)	The Result of the Statistical Test
Age	X ± SD (Me)	28.9 ± 3.7 (29.0)	29.9 ± 5.6 (29.0)	NS (*p* = 0.18) /#
Place of residence	City	100 (64.94%)	139 (85.28%)	*p* = 0.00005 *
	Rural area	54 (35.06%)	24 (14.72%)	
Education	Primary	0 (0.00%)	17 (10.43%)	*p* < 0.00001 *
	Secondary	58 (37.66%)	66 (40.49%)	
	Higher	96 (62.34%)	80 (49.08%)	
Type of occupation	Intellectual	98 (63.64%)	78 (47.85%)	*p* = 0.02 *
	Manual	30 (19.48%)	44 (26.99%)	
	Not working	26 (16.88%)	41 (25.15%)	
Marital status	Single	12 (7.79%)	17 (10.43%)	NS (*p* = 0.53) *
	In a relationship	142 (92.21%)	146 (89.57%)	
Financial status	Bad @	2 (1.30%)	1 (0.61%)	NS (*p* = 0.62) *
	Average @	40 (25.97%)	37 (22.70%)	
	Good	92 (59.74%)	99 (60.74%)	
	Very good	20 (12.99%)	26 (15.95%)	

X—mean value; SD—standard deviation; Me—median; NS—statistically non-significant difference; *p*—level of statistical significance; *—CHI^2^ test result with Yates correction; /#—Mann–Whitney U test result. (@) in the following parts of the analysis, these two categories of “material situation” are combined into one.

**Table 2:** Characteristics of the study group—clinical variables.

Factor	Category	“Pregnancy” Group (*n* = 154; 100%)	“Postpartum” Group (*n* = 163; 100%)	The Result of the Statistical Test
Week of pregnancy (for the “pregnancy” group on the day of the test, for the “Postpartum” group at the time of delivery)	X ± SD (Me)	36.1 ± 1.8 (36.0)	38.4 ± 2.1 (38.0)	*p* < 0.00001 /#
Number of pregnancies	Primipara	106 (68.83%)	76 (46.63%)	*p* = 0.0001 *
	Multipara	48 (31.17%)	87 (53.37%)	
Course of delivery	Natural delivery	---	89 (54.60%)	---
	C–section	---	74 (45.40%)	
Course of pregnancy/without complications	No	40 (25.97%)	55 (33.74%)	NS (*p* = 0.17) *
	Yes	114 (74.03%)	108 (66.26%)	
Psychiatric treatment before current pregnancy (in the past)	No	132 (85.71%)	158 (96.93%)	*p* = 0.0007 *
	Yes	22 (14.29%)	5 (3.07%)	
Prolonged periods of anxiety and sadness before the current pregnancy	No	130 (84.42%)	153 (93.87%)	*p* = 0.01 *
	Yes	24 (15.58%)	10 (6.13%)	
Diagnosed depressive disorders before current pregnancy	No	142 (92.21%)	154 (94.48%)	NS (*p* = 0.56) *
	Yes	12 (7.79%)	9 (5.52%)	
Support from husband/partner/family during pregnancy/postpartum	No	16 (10.39%)	8 (4.91%)	NS (*p* = 0.10) *
	Yes	138 (89.61%)	155 (95.09%)	

X—mean value; SD—standard deviation; Me—median; NS—statistically non-significant difference; *p*—level of statistical significance; *—CHI^2^ test result with Yates correction; /#—Mann–Whitney U test result.

**Table 3:** The average score for EPDS obtained in study groups. The mean ± standard deviation and median are listed.

Scale	“Pregnancy” Group (*n* = 154)	“Postpartum” Group (*n* = 163)	Mann–Whitney’s U TEST
EPD	10.1 ± 6.4 (9.0)	8.2 ± 5.7 (6.0)	*p* = 0.005

**Table 4:** Distributions relative to accepted threshold values of indicators (TOTAL scores) of EPDS in study groups.

Scale	Range of Points	“Pregnancy” Group (*n* = 154)	“Postpartum” Group (*n* = 163)	CHI^2^ Test with Yates Correction
EPDS	0–9	80 (51.95%)	108 (66.26%)	*p* = 0.03
	10–13	34 (22.08%)	28 (17.18%)	
	14 and above	40 (25.97%)	27 (16.56%)	

**Table 5:** Distribution of responses to EPDS question No. 10 (“*Were there times when you thought about hurting yourself?*”) in study groups.

Reply	“Pregnancy” Group (*n* = 154; 100%)	“Postpartum” Group (*n* = 163; 100%)	CHI^2^ Test with Yates Correction
Never	136 (88.31%)	134 (82.21%)	NS (*p* = 0.17)
Other responses (rarely or sometimes or yes, quite often)	18 (11.69%)	29 (17.79%)	

**Table 6:** Numbers of affirmative responses (thoughts of self-harm) to question 10 of the EPDS scale in the study groups with respect to sociodemographic and obstetric factors.

Factor	Categories	Group “Pregnancy	“Postpartum” Group	Comparison Between Groups
Place of residence	City	14 (14.00%)	19 (13.67%)	NS (*p* = 0.91)
	Rural area	4 (7.41%)	10 (41.67%)	*p* = 0.0009
	CHI^2^ test in the group	NS (*p* = 0.34)	*p* = 0.003	
Education	Primary	---	1 (5.88%)	---
	Secondary	10 (17.24%)	8 (12.12%)	NS (*p* = 0.58)
	Higher	8 (8.33%)	20 (25.00%)	*p* = 0.005
	CHI^2^ test in the group	NS (*p* = 0.16)	*p* = 0.04	
Type of occupation	Intellectual	12 (12.24%)	7 (8.97%)	NS (*p* = 0.65)
	Manual	2 (6.67%)	13 (29.55%)	*p* = 0.03
	Not working	4 (15.38%)	9 (21.98%)	NS (*p* = 0.73)
	Comparison in the group	NS (*p* = 0.57)	*p* = 0.01	
Marital status	Single	2 (16.67%)	6 (35.29%)	NS (*p* = 0.49)
	In a relationship	16 (11.27%)	23 (15.97%)	NS (*p* = 0.35)
	CHI^2^ test in the group	NS (*p* = 0.93)	NS (*p* = 0.10)	
Financial status	Bad or average	8 (19.05%)	9 (23.68%)	NS (*p* = 0.82)
	Good	10 (10.87%)	16 (16.16%)	NS (*p* = 0.39)
	Very good	0	4 (15.38%)	NS (*p* = 0.19)
	CHI^2^ test in the group	NS (*p* = 0.12)	NS (*p* = 0.54)	
Parity	Primiparas	8 (7.55%)	19 (25.00%)	*p* = 0.002
	Multiparas	10 (20.83%)	10 (11.49%)	NS (*p* = 0.23)
	CHI^2^ test in the group	*p* = 0.04	*p* = 0.04	
Child birth (natural, C-section)	Natural delivery		16 (17.98%)	---
	C–section		13 (17.57%)	---
	CHI^2^ test in the group		NS (*p* = 0.89)	
Course of pregnancy without complications	No	6 (15.00%)	15 (27.27%)	NS (*p* = 0.24)
	Yes	12 (10.53%)	14 (12.95%)	NS (*p* = 0.72)
	CHI^2^ test in the group	NS (*p* = 0.64)	*p* = 0.04	
Psychiatric treatment before current pregnancy	No	12 (9.09%)	28 (17.72%)	*p* = 0.05
	Yes	6 (27.27%)	1 (20.00%)	NS (*p* = 0.82)
	CHI^2^ test in the group	*p* = 0.04	NS (*p* = 0.64)	
Prolonged periods of anxiety and sadness before pregnancy	No	8 (6.15%)	28 (18.30%)	*p* = 0.004
	Yes	10 (41.67%)	1 (10.00%)	NS (*p* = 0.16)
	CHI^2^ test in the group	*p* < 0.00001	NS (*p* = 0.81)	
Diagnosed depressive disorder before pregnancy	No	14 (9.86%)	25 (16.23%)	NS (*p* = 0.18)
	Yes	4 (33.33%)	4 (44.44%)	NS (*p* = 0.95)
	CHI^2^ test in the group	*p* = 0.05	NS (*p* = 0.09)	
Support from husband/partner/family during pregnancy/postpartum	No	8 (50.00%)	6 (75.00%)	NS (*p* = 0.46)
	Yes	10 (7.25%)	23 (14.84%)	NS (*p* = 0.07)
	CHI^2^ test in the group	*p* < 0.00001	*p* = 0.0001	

## Data Availability

The datasets analyzed during the current research are available from the corresponding author on reasonable request.
